# In-Situ Testing of the Thermal Diffusivity of Polysilicon Thin Films

**DOI:** 10.3390/mi7100174

**Published:** 2016-10-01

**Authors:** Yi-Fan Gu, Zai-Fa Zhou, Chao Sun, Wei-Hua Li, Qing-An Huang

**Affiliations:** Key Laboratory of MEMS of the Ministry of Education, Southeast University, Nanjing 210096, China; yfgu@seu.edu.cn (Y.-F.G.); sun_chaos@hotmail.com (C.S.); liwh@seu.edu.cn (W.-H.L.); hqa@seu.edu.cn (Q.-A.H.)

**Keywords:** thermal diffusivity, process control monitoring (PCM), in-situ testing, thin film

## Abstract

This paper presents an intuitive yet effective in-situ thermal diffusivity testing structure and testing method. The structure consists of two doubly clamped beams with the same width and thickness but different lengths. When the electric current is applied through two terminals of one beam, the beam serves as thermal resistor and the resistance *R*(*t*) varies as temperature rises. A delicate thermodynamic model considering thermal convection, thermal radiation, and film-to-substrate heat conduction was established for the testing structure. The presented in-situ thermal diffusivity testing structure can be fabricated by various commonly used micro electro mechanical systems (MEMS) fabrication methods, i.e., it requires no extra customized processes yet provides electrical input and output interfaces for in-situ testing. Meanwhile, the testing environment and equipment had no stringent restriction, measurements were carried out at normal temperatures and pressures, and the results are relatively accurate.

## 1. Introduction

In order to further develop micro electro mechanical systems (MEMS) fabrication and achieve its improved integration into integrated circuit (IC) fabrication, process control monitoring (PCM) techniques, which are widely applied in IC fabrication to obtain detailed information about the process, should also be applied with MEMS manufacturing. Unfortunately, traditional PCM techniques have been proven incompatible or unable to cover all the new characteristics MEMS brings in. Therefore, it has been necessary to develop new PCM techniques to test new properties of interest, including mechanical, electrical, magnetic, and thermal properties. The thermal design of devices is important for enhancing performance and reliability. However, traditional methods used in measuring thermal diffusivity fail to meet the requirements of in-situ testing thin film materials in MEMS. Owing to the significance of the property, extensive studies have been conducted, and several experimental techniques have been developed to measure the thermal diffusivity of thin film materials [1,2,3,4].

Hatta et al. introduced an Alternating Current (AC) calorimeter method to measure thermal diffusivity [5]. In this method, an AC thermal energy is supplied to the sample membrane and mask via modulated light irradiation. By measuring the attenuation and phase lagging of temperature waves across a certain distance of the sample, thermal diffusivity of the sample can be obtained. In this measurement as well as some other similar counterparts [6,7], an external light source is required to supply the thermal pulses, and the measuring of temperature waves relies on thermocouples. The overall system is rather complicated and is difficult to integrate into the present PCM systems.

Zhang et al. presented a method to acquire thermal diffusivity using a micro bridge [8]. A heater and a sensor were attached to a bridge. The bases which the bridge was clamped to were regarded as heat sinks. By forcing a specified current to the heater, and applying the phase-shift method, the amplitude method, or the heat-pulse method, thermal diffusivity could be acquired. In this measurement, the so-called micro-devices was fabricated by sputtering a tungsten layer on top of the bridge, then lithographed and etched to the predefined geometry. Obviously, extra processes were required, which increased the fabrication difficulty and cost. Moreover, the stress introduced by the extra processes might have affected the thermal properties of the thin film, causing the result to be deviated from the reality.

In the present work, thermal diffusivity was in-situ tested using groups of doubly clamped beams. The beams were fabricated with materials of interest and served as thermal resistors themselves. By changing the applied current through the beams and recording the consequential change of voltage, thermal diffusivity could be acquired by substituting the acquired data into a carefully derived thermodynamic model. Compared with the previous studies, the presented method in this paper satisfies the requirement of in-situ testing, and no extra process is needed. The results prove to be robust and reliable.

## 2. Theory

### 2.1. Heat Transfer

As is known, heat is mainly transferred through three methods: heat conduction, thermal convection, and thermal radiation [9]. According to Fourier’s Law, 1-D heat conduction can be described as

(1) $$q_{cond}=\frac{Q}{A}=-\lambda \frac{dT}{dx},$$

in which *q_cond_* is the heat flux induced by heat conduction, *Q* the power of heat flow, *A* the cross-sectional area, λ the material’s thermal conductivity, and *dT/dx* the temperature gradient. Newtonian cooling theory states that thermal convection between a surface and fluid can be described as

(2) $$q_{conv}=h_{c}\cdot \Delta T,$$

in which *q_conv_* is the heat flux induced by thermal convection, *h_c_* the heat transfer coefficient, and Δ*T* the temperature difference. Meanwhile, the Stefan–Boltzmann Law provides radiant intensity:

(3) $$q_{rad}=\varepsilon \sigma \cdot \left(T^{4}-T_{0}^{4}\right),$$

in which *q_rad_* is the heat flux induced by thermal radiation. ε is the emissivity of the material, which is dependent on various factors and is reported ranging from approximately 0.4 to 0.95 for polysilicon [10,11]. Radiation-caused dissipation is negligible in the present work, as the testing environment is normal temperature and pressure. Therefore, emissivity is relatively insignificant and is reasonable to be taken as 0.9 in the present study. σ is the Stefan–Boltzmann constant, with a value of 5.670373 × 10^−8^ W/(m^2^·K^4^). The abovementioned heat transfer mechanism is essential to various scenarios in MEMS, including microheater-based chemosensors [12,13], high temperature applications [14,15], heat sinks in power devices [16,17], energy harvesting applications [18,19], and so forth. It is also the basis of the following analysis of the presented in-situ testing structure.

### 2.2. In-Situ Testing Structure and Thermodynamic Analysis

**Figure 1:**
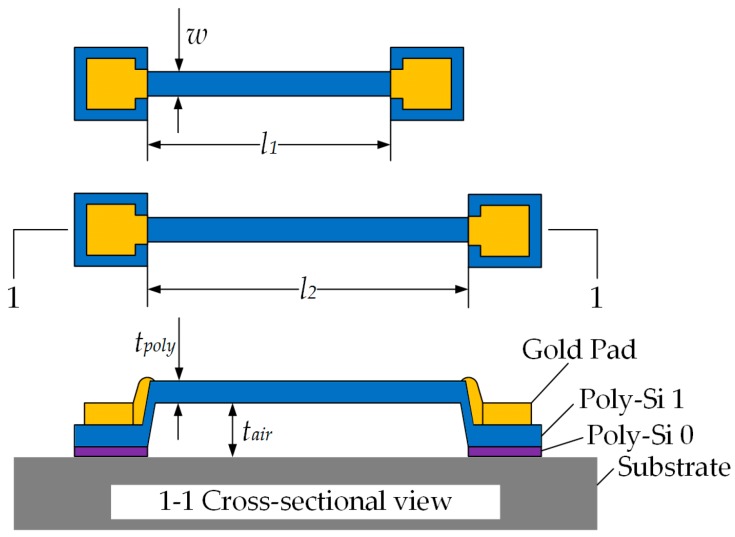
Top and cross-sectional view of the configuration of the in-situ testing structure. The presented structure was fabricated by Central Semiconductor Manufacturing Corporation (CSMC) using complementary metal-oxide semiconductor (CMOS) compatible processes. The beam fabricated with polysilicon stood on another structured layer “Poly-Si 0” and was capped with gold pads, which served as an electrical I/O.

The schematic of the in-situ testing structure is shown in Figure 1. The structure consisted of two doubly clamped beams with the same width, *w*, and thickness, *t_poly_*, but different lengths, *l*_1_ and *l*_2_. The beams were fabricated with the material to be tested, and the material had to be electrically conductive, which was heavily doped polysilicon in the present study. The pads were fabricated with gold. The two beams were fabricated simultaneously, so the material properties should have been identical, while the process variations were considered quasi-identical, provided that the substrate being considered was an ideal heat sink, meaning that the heat transferred from beam to substrate can sink quickly without influencing the substrate temperature.

When a constant current *I*_0_ was forced into a beam through correspondent pads, temperature *T* of the beam rose due to the joule heat, and the resistance *R* of the beam changed simultaneously as a function of temperature. After the heat balance was established, temperature *T* is stabilized and thus is resistance. The heat balance meant that the generated thermal energy equaled the dissipated thermal energy. Then, the heating of the beam was stopped by applying a rather small constant current *I_t_* instead of *I*_0_. The purpose of applying the current *I_t_* was to measure the resistance after the heating was stopped. Obviously, the temperature *T* would decrease as the generation of joule heat dropped, until a new heat balance was built. The dissipation of thermal energy, which can be characterized as the falling of temperature, was determined by the geometric parameters as well as thermal properties, such as thermal diffusivity. While the geometric parameters were already known, the thermal properties could be acquired by measuring and analyzing the descending curve of the temperature.

**Figure 2:**
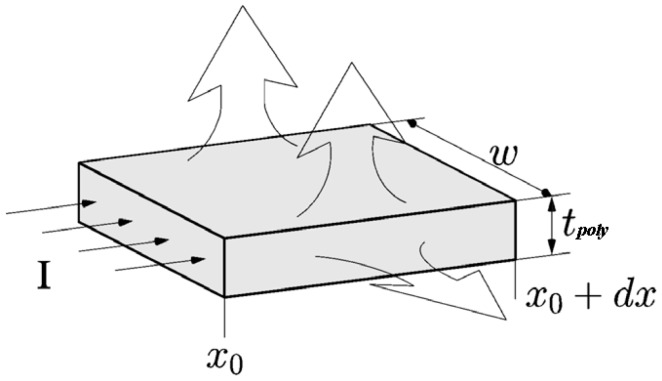
Volume element of the beam. Heat dissipates from the top, bottom, and two side surfaces to the environment. Current flows in from the cross-sectional area at *x*_0_ and flows out at *x*_0_ + *dx*.

Consider a volume element with width *w*, thickness *t_poly_*, and length *dx*, as shown in Figure 2. The heat balance equation can be described as follows:

(4) $$-\lambda \frac{\partial T\left(x_{0}\right)}{\partial x}+\frac{I^{2}}{A}\cdot \rho_{0}\left[1+\xi \left(T-T_{0}\right)\right]\cdot dx=-\lambda \frac{\partial T\left(x_{0}+dx\right)}{\partial x}+Q_{out}\prime +\rho_{poly}C\cdot \frac{\partial T}{\partial t}dx,$$

in which λ is the thermal conductivity, *A = w·t_poly_* the cross-sectional area, ρ_0_ the resistivity of the beam material at temperature *T*_0_, ξ the linear temperature coefficient of the resistivity, ρ*_poly_* the density, and *C* the specific heat capacity of the material. *Q_out_′ = Q_conv_ + Q_rad_* consist of the thermal convection and radiation part:

(5) $$Q_{conv}=Q_{conv}\prime +Q_{conv-sub}=h_{c}\left(w+2t_{poly}\right)\left(T-T_{0}\right)\cdot dx+\frac{S}{R_{T}}w\left(T-T_{0}\right)\cdot dx,$$

(6) $$Q_{rad}\approx \varepsilon \sigma \left(w+2t_{poly}\right)dx\cdot 4T_{0}^{3}\left(T-T_{0}\right),$$

in which *Q_conv_′* includes the convection of the top and two side surfaces with the environment, while *Q_conv-sub_* indicates the thermal convection of the bottom surface with the substrate and the air gap in between. *S = (t_poly_/w)·(*2*t_air_/t_poly_* + 1) + 1 is the thermal convection shape coefficient [20], in which *t_air_* is the thickness of the air gap. *R_T_ = t_air_*/λ*_air_* is the thermal resistance of the air gap. Substituting thermal conductivity λ = α·ρ*_poly_·C* into Equation (4) and simplifying it gives the partial differential equation form of transient heat transfer equation:

(7) $$\frac{\partial^{2}T}{\partial x^{2}}=\frac{1}{\alpha }\cdot \frac{\partial T}{\partial t}+\frac{K}{\lambda }\left(T-T_{0}\right)-\frac{I^{2}}{\lambda A}\cdot \rho_{0},$$

(8) $$K=h_{c}\left(w+2t_{poly}\right)+\frac{S}{R_{T}}w+\varepsilon \sigma \left(w+2t_{poly}\right)\cdot 4T_{0}^{3}-\frac{I^{2}}{A}\cdot \rho_{0}\xi .$$

The transient temperature distribution *T*(*x, t*) can be acquired by solving the partial differential Equation (7) [21]. Therefore, the resistance of the beam can be expressed as an integration of the temperature distribution, while the relationship between thermal diffusivity α and a testable electrical parameter, namely the resistance of the beam *R* [22], is obtained as

(9) $$R\left(t\right)=\int_{0}^{l}R_{0}\left[1+\xi \left(T\left(x,t\right)-T_{0}\right)\right]dx=c_{0}+c_{1}\exp\left(-\frac{t}{\tau }\right),$$

(10) $$c_{0}=R_{0}+R_{0}\cdot \xi \cdot \left(\frac{I^{2}\cdot \rho_{0}}{A\cdot \lambda \cdot G}-\frac{2I^{2}\cdot \rho_{0}}{A\cdot \lambda \cdot G^{\frac{3}{2}}\cdot l}\cdot \tanh(\frac{\sqrt{G}}{2}l)\right),$$

(11) $$c_{1}=\frac{4I^{2}\cdot \rho_{0}}{A\cdot \lambda \cdot G\cdot \pi }\left(-\frac{2}{\pi }+\frac{2\pi }{Gl^{2}+\pi^{2}}\right),$$

(12) $$\tau =\frac{l^{2}}{\alpha \cdot \left(Gl^{2}+\pi^{2}\right)},$$

(13) $$G=\frac{h_{c}\left(w+2t_{poly}\right)+\frac{S}{R_{T}}w+4\varepsilon \sigma T_{0}^{3}\left(w+2t_{poly}\right)-I^{2}\cdot \rho_{0}\cdot \xi }{\lambda \cdot A},$$

in which *c*_0_, *c*_1_, and *G* are α-independent coefficients, and τ the time constant of the beam. *R*_0_ is measured at environment temperature *T*_0_, while *R*(*t*) is sampled and recorded throughout the testing process, and the final heat balance gives *R*_∞_.

**Figure 3:**
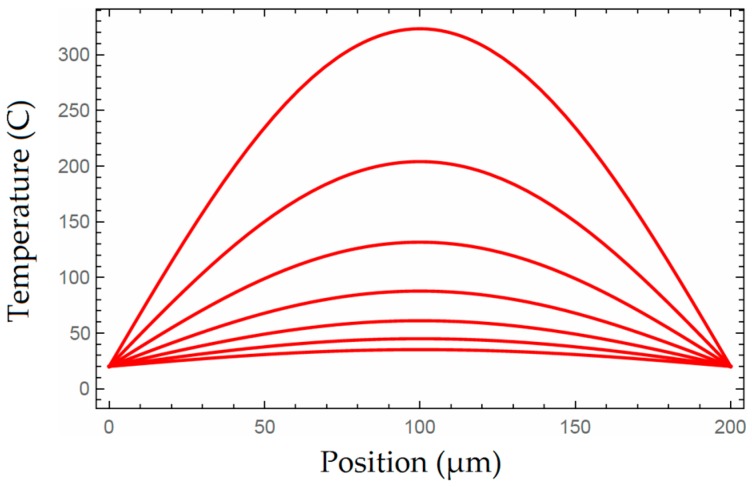
Temperature distribution and temperature decrease after heating stopped.

Figure 3 illustrates the temperature distribution on a heated beam. The multiple curves indicate temperature distribution at specific moments after the stop of heating. As can be seen from the figure, the distribution has a parabolic shape across the length of the beam, meaning that the highest temperature appears at the center of a beam. Meanwhile, as the time intervals between the moments are taken to be identical, it is obvious that temperature decreases slower as it approaching room temperature. Furthermore, when applying the same amount of current across the beams, a longer beam means a higher peak temperature as well as a slower decrease in temperature after heating stops, which is due to greater resistance across the beam and a larger time constant according to Equation (12).

The Equation (9) can be transformed into

(14) $$\ln\left(\frac{R\left(t\right)-R_{\infty }}{R_{0}}\right)=\ln\left(\frac{B}{A+B}\right)-\frac{t}{\tau }=c-\frac{t}{\tau },$$

in which *c* is an α-independent constant. As can be seen, the logarithmic function of *R* is linear with time *t*, with a slope of −1/τ. Therefore, the time constant τ of a beam can be easily acquired by substituting recorded resistance values into Equation (14) and performing linear fitting. Substitute the acquired time constants and lengths of the beams into Equation (12) and eliminate parameter *G*, the only left unknown parameter is thermal diffusivity, which can be easily obtained as

(15) $$\alpha =\left(\frac{1}{\tau_{1}}-\frac{1}{\tau_{2}}\right)/\pi^{2}\left(\frac{1}{l_{1}^{2}}-\frac{1}{l_{2}^{2}}\right),$$

in which the subscripts “1” and “2” represent two beams with different lengths accordingly.

## 3. Experiment and Results

### 3.1. Fabrication

**Figure 4:**
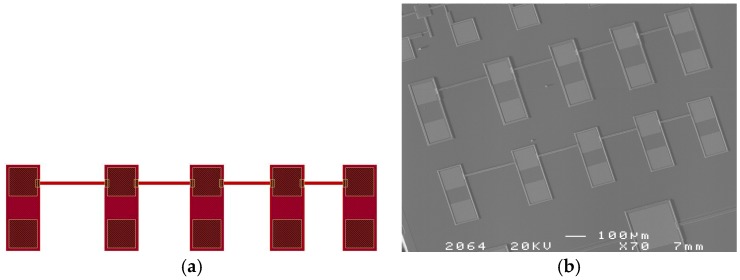
(**a**) Layout of the in-situ testing structure. The beams from left to right are 250 μm, 200 μm, 180 μm, 150 μm in length accordingly. (**b**) SEM photo of the testing structure. Two sets of structures are included in the photo.

Layout of the in-situ testing structure is shown in Figure 4a. The structure consists of 4 beams with different length. Each beam corresponds to 4 pads, which are used to apply electrical excitation and extract responses. The samples were fabricated by CSMC (CSMC stands for Central Semiconductor Manufacturing Corporation, which is a subsidiary of China Resources Microelectronics Limited and is one of the largest 6-inch/8-inch foundries in Mainland China) using a CMOS compatible process. The adopted processes are identical to that of CSMC’s commercial products. Firstly, a 6-inch <110> silicon wafer coated with silicon nitride was used as a substrate. A layer of polysilicon “Poly-Si 0” with a thickness of 0.3 μm was deposited upon the substrate using low-pressure chemical vapor deposition (LPCVD), then etched and heavily doped. Actually, “Poly-Si 0” is not necessary in the presented testing method, and was only fabricated to follow standard procedure. Then, a sacrificial layer with a thickness of 2 μm was deposited and etched, so that the following deposition of the 2-μm-thick “Poly-Si 1” could resulting in having some parts of the polysilicon sitting right on the “Poly-Si 0” while other parts lay on the sacrificial layer. Finally, by heavily doping “Poly-Si 1” and etching according to the designed patterns, the testing structures were fabricated. Evaporated gold film was attached to “Poly-Si 1” and shaped as metal caps on the electrodes using a peel-off technique. While “Poly-Si 1” was heavily doped and conductive, the metal caps were not essential parts of the testing structures, which only served to enhance the electrical connectivity when applying the probes during testing. Geometric parameters and material properties of the sample beams are listed in Table 1. The SEM photo of the fabricated structures is shown in Figure 4b.

### 3.2. Experiment

**Figure 5:**
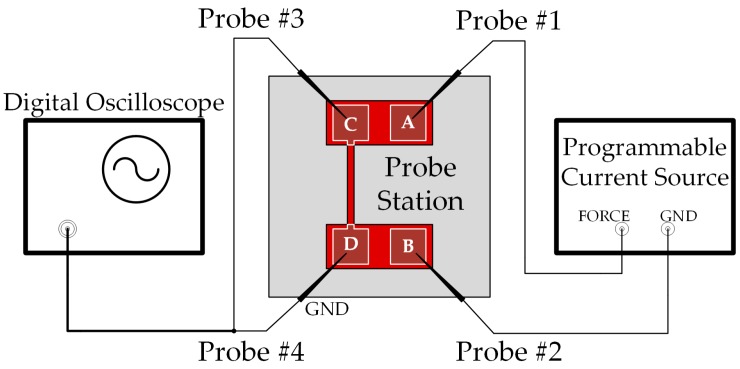
Schematic diagram of thermal diffusivity in-situ testing circuit.

Four probes were positioned on the pads accordingly as shown in Figure 5. The pad A and B, which were away from the beam, were used to apply current through the probes. The pad C and D were connected to a digital oscilloscope. In the present study, Keithley 4200-SCS Parameter Analyzer (Keithley Instruments, Solon, OH, USA) was used as a programmable current source, and Agilent InfiniiVision 3032A Digital Oscilloscope (Agilent Technologies, Santa Clara, CA, USA) was used to characterize the relationship between voltage *V*(*t*) and time *t*, and the resistance *R*(*t*) could be obtained by dividing the voltage by the current applied.

**Figure 6:**
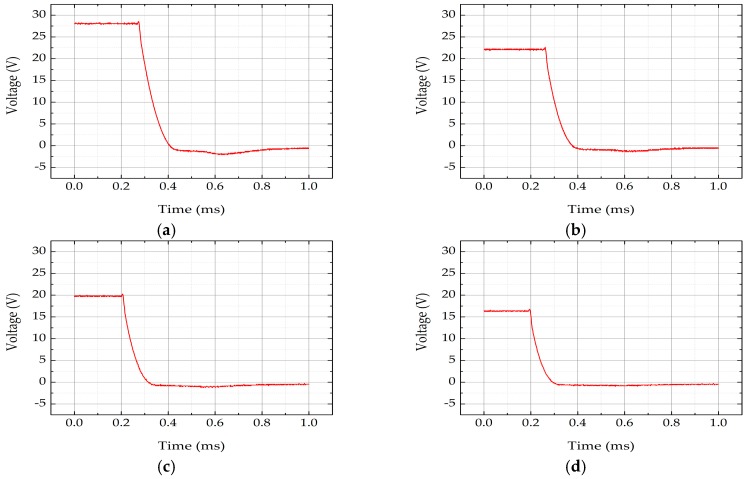
The falling edge of voltage across beams recorded by oscilloscope: (**a**) *l*_1_ = 250 μm; (**b**) *l*_2_ = 200 μm; (**c**) *l*_3_ = 180 μm; (**d**) *l*_4_ = 150 μm.

**Figure 7:**
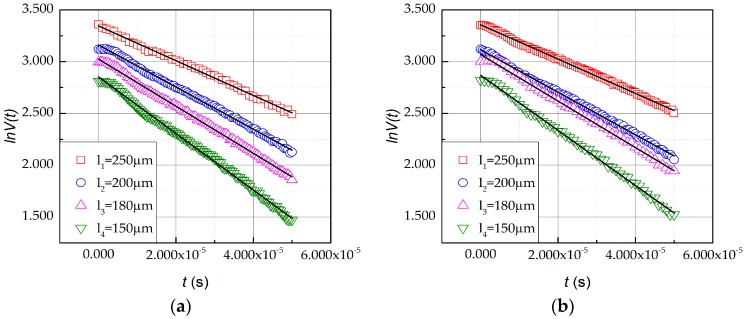

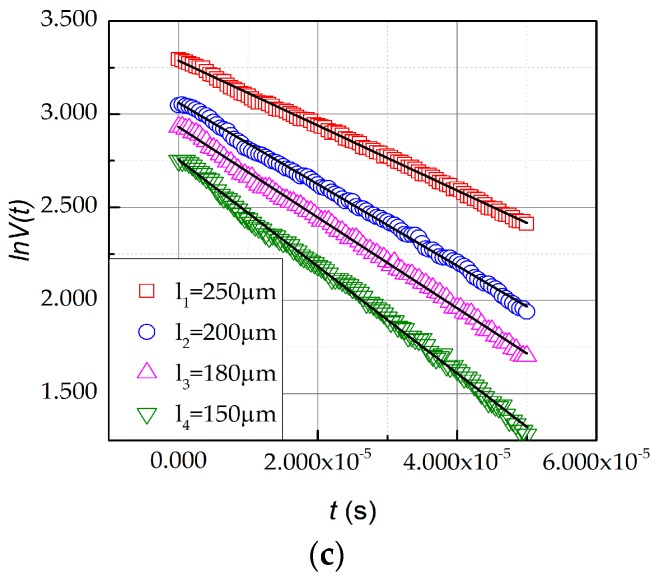
The fitting of linear relationship between ln*V*(*t*) and *t* of three sets of beams: (**a**) Fitting results of Set #1; (**b**) Fitting results of Set #2; (**c**) Fitting results of Set #3.

A constant current *I*_0_ = 2 mA was applied to the beam, and the temperature of the beam rose due to the joule heat. The stabilizing period usually takes no more than 1 ms. Then, the applied current decreases to *I*_t_ = 0.01 mA, resistance changes as the temperature drops, and voltage measured by oscilloscope consequently changes. In the present study, considering the current *I*_t_ was taken small enough, an approximation of *R_0_* ≈ *R_∞_* was made, and *V_∞_* was negligible compared with *V*(*t*), which yielded:

(16) $$\ln\left(\frac{R\left(t\right)-R_{\infty }}{R_{0}}\right)\approx \ln\left(\frac{V\left(t\right)-V_{\infty }}{V_{\infty }}\right)\approx \ln V\left(t\right)=c\prime -\frac{t}{\tau },$$

in which *c*′ is an α-independent constant other than *c*. The falling edges of *V*(*t*) recorded by oscilloscope are exhibited in Figure 6, indicating that the beam material had a positive temperature coefficient ξ. Choosing a proper section of the curve, every recorded data point from said section is used to fit into Equation (14). In the present study, 3 sets of beams were tested accordingly, and the respective curve section chosen began with the start of the falling edge and lasted for 50 μs. The relation between ln*V*(*t*) and *t* is plotted in Figure 7. The slope of the line fitted by the points is the reciprocal of time constant *τ* of the beam. The fitted values 1/τ of the tested sets are listed in Table 2. By substituting pairs of *τ* and respective *l* into Equation (15), thermal diffusivity α was acquired.

### 3.3. Results

For each set with four beams in different lengths, six possible combinations were calculated and averaged as listed in Table 3. As shown in Table 2 and Table 3, the time constant of the tested beams showed good consistency that the deviation was below 3%, while the thermal diffusivity was below 5%. The average thermal diffusivity of the three tested sets is 41.43 mm^2^/s.

The thermal diffusivity of heavily doped LPCVD polysilicon films was reported to be 17 mm^2^/s by Mastrangelo and Muller [22], while single crystal silicon was reported as 82–95 mm^2^/s [5]. According to many other reports [23,24,25,26,27], heavily doped polysilicon has a thermal diffusivity ranging from 21.43 mm^2^/s to 36.56 mm^2^/s. Thermal characteristics can vary significantly from small differences in processes and geometrical settings. While the dopant concentration was similar, at the level of approximately 10^19^–10^20^ cm^−3^, the larger result of the present work 41.43 mm^2^/s can be attributed to the larger thickness of the polysilicon thin film used in the present study, which caused the grains in the film to be larger. Meanwhile, it is obvious that thermal diffusivity of doped polysilicon should be much lower than single crystal silicon due to ubiquitous grain boundaries and defects within. Therefore, the results of the present work are rational and reliable.

A recently published paper by Nishimura et al. [28] proposes a method that requires only the fabrication of a thin film on a prepared sensor using a relatively simple measuring system and provides robust results. However, the sensor was prepared before the fabrication of the thin film, thus introducing extra cost. The presented in-situ testing structures could be fabricated along with the purposed products as subsidiary, and could even be fabricated on a scribe line of a wafer, which would waste no footprint on the dies. Other researchers [29,30,31] have recently presented methods that are capable of measuring thermal properties with high precision; however, the measurements are carried mainly in vacuum chambers, which fail to meet the criteria of the PCM technique. Though these inspiring methods have advantages in various aspects, the proposed method is more applicable and flexible considering the scenario of integrating MEMS with conventional IC fabrication, as well as the monitoring systems.

## 4. Conclusions

This paper presented a feasible in-situ thermal diffusivity testing structure and testing method. It is compatible with PCM in IC fabrication, benefiting from its capability of applying excitation and extracting feedback completely electrically.

The development of such a MEMS-friendly PCM technique is necessary for the integration of MEMS and IC fabrication. However, the method has the drawback that it is not applicable to nonconductive materials or materials with very high resistance. Modern MEMS fabrication processes are usually capable of manufacturing multilayered structures; therefore, nonconductive thin films could be stacked over conductive thin films. With a few modifications of the present thermodynamic model, thermal diffusivity of nonconductive materials could also be easily acquired.

## Figures and Tables

**Table 1:** Geometric parameters and material properties of the sample beams.

Width *w* (μm)	Thickness *t_poly_* ^1^ (μm)	Length *l* (μm)				Air GAP Thickness *t_air_* ^1^ (μm)	Sheet Resistance *R_S_* (Ω)	Young’s Modulus *E* (GPa)	Residual Stress σ (MPa)
10	2	*l*_1_	*l*_2_	*l*_3_	*l*_4_	2	9.5	115.8	−5.5
		250	200	180	150				

^1^ The thickness of beams and air gap is constant according to the process.

**Table 2:** The reciprocal of time constant (1/τ).

Length *l* (μm)	Set #1 (s^−1^)	Set #2 (s^−1^)	Set #3 (s^−1^)
*l*_1_ = 250	1.721 × 10^4^	1.747 × 10^4^	1.741 × 10^4^
*l*_2_ = 200	2.140 × 10^4^	2.150 × 10^4^	2.185 × 10^4^
*l*_3_ = 180	2.424 × 10^4^	2.421 × 10^4^	2.431 × 10^4^
*l*_4_ = 150	2.852 × 10^4^	2.846 × 10^4^	2.865 × 10^4^

**Table 3:** Averaged thermal diffusivity of each set of beams.

Thermal Diffusivity	Set #1	Set #2	Set #3	Averaged Value
α (mm^2^/s)	42.23	40.87	41.20	41.43
